# Pandemic-era increases in late-stage pediatric cancer diagnoses, 2020-2022

**DOI:** 10.1093/jncics/pkag067

**Published:** 2026-06-22

**Authors:** Todd Burus, Jason Semprini

**Affiliations:** Markey Cancer Center, University of Kentucky, Lexington, KY, United States; Department of Internal Medicine, College of Medicine, University of Kentucky, Lexington, KY, United States; Department of Public Health, Des Moines University College of Health Sciences, West Des Moines, IA, United States

## Abstract

COVID-19 pandemic-related disruptions may have altered pediatric cancer detection in the United States. Using the National Childhood Cancer Registry, we compared observed incidence rates among individuals aged 0-17 years during 2020-2022 with expected rates with 95% credible intervals (CrIs) derived from pre-pandemic trends. Rates were evaluated overall and by race/ethnicity, cancer site, and stage-at-diagnosis. Among 27 996 cancers diagnosed in 2020-2022, total incidence was 17.44 per 100 000, similar to the expected rate of 17.39 (95% CrI = 16.80-17.98). Annual patterns showed an incidence reduction in 2020, followed by modestly higher-than-expected rates in 2021-2022. Pandemic-era late-stage incidence was significantly higher than expected, however (10.58 vs 10.19 per 100 000). Overall, pediatric cancer incidence during the COVID-19 pandemic matched expectations, but late-stage diagnoses increased, underscoring the need to restore timely evaluation and maintain diagnostic access during health-system disruptions.

While pediatric COVID-19 mortality was low in the United States, other pandemic-associated impacts on children remain a concern.^1^^,^^2^ Building upon prior work documenting changes in pediatric cancer burden during 2020-2021,^3^^,^^4^ we aimed to investigate changes in pediatric cancer incidence across the first 3 years of the pandemic. We compared observed pediatric cancer incidence rates for 2020-2022 with expected rates by sociodemographic factors, cancer site, and stage.

Data for this study were obtained from the National Childhood Cancer Registry (NCCR)—a specialized collection of information on pediatric, adolescent, and young adult cancers that includes 25 states and covers approximately 73% of the US population aged 0-39 years. The NCCR database—part of the National Cancer Institute’s Childhood Cancer Data Initiative—relies on submissions to the North American Association of Central Cancer Registries Cancer in North America dataset and is made available for research purposes through the SEER*Stat platform. We identified malignant cancer cases among individuals aged 0-17 years using SEER*Stat version 9.0. Annual case counts for 2005-2022 were stratified by single year of age. We extracted additional information on race/ethnicity (Hispanic, non-Hispanic Black, non-Hispanic White, other non-Hispanic races), cancer site, and stage at diagnosis (early, late, other). Race and ethnicity were identified according to the SEER Race and origin recode variable. “Other non-Hispanic races” combined non-Hispanic American Indian/Alaska Native, Asian or Pacific Islander, and unknown races due to small counts. Cancer sites were identified according to the *International Classification of Childhood Cancer*, Third Edition (ICCC-3) site groups: leukemias; lymphomas; central nervous system (CNS) tumors; neuroblastomas; retinoblastoma; renal tumors; hepatic tumors; bone tumors; soft tissue sarcomas; germ cell tumors; other epithelial neoplasms and melanomas; and other malignant neoplasms.^5^ Stage at diagnosis was based on the SEER program’s Summary Stage (2004+) variable, with early stage defined as localized disease, late stage including both regional and distant stages, and other defined as unknown stage, stage not available, or blank.

We estimated expected annual and aggregate (2020-2022) age-adjusted incidence rates with 95% credible intervals (CrIs) from 2005 to 2019 case counts using Bayesian age-period-cohort (BAPC) models. BAPC models were fit to annual, single-age case counts to model incidence rates as a Poisson process with random effects for age, period, and birth cohort (using second-order random walk priors), along with an independent and identically distributed random effect to account for overdispersion.^6^ Variance for all random effect parameters were specified using log-gamma priors with shape parameter *a* = 1 and rate parameter *b* = 0.00005. Separate models were fit for all combinations of site-, race and ethnicity-, and stage-specific case counts considered. Additional details on the modeling procedure and model validation can be found in Burus et al.^7^ Model diagnostic plots for all overall and stage-specific models are included in the Supplementary materials (Figures S1-S3). Observed rates were considered significantly different from expected if they fell outside the 95% CrI, indicating that there was a <5% probability of the observed rate having occurred based on pre-pandemic trends. This study was deemed exempt from review by the University of Kentucky Institutional Review Board. We followed STROBE reporting guidelines.

There were 27 996 pediatric cancer cases reported in 2020-2022, with 8628 (30.8%) among Hispanic individuals, 3125 (11.2%) among non-Hispanic Black individuals, 13 636 (48.7%) among non-Hispanic White individuals, and 2607 (9.3%) among individuals of other non-Hispanic races. Leukemias were the most frequently diagnosed cancers (28.2%), followed by CNS tumors (16.7%). Most cases were diagnosed at a late stage (60.7%) (Table 1).

**Table 1.** Observed vs expected pediatric cancer incidence rates for the COVID-19 pandemic.

Group	Count, *N* (%)	**Observed rate** ^a^	Expected rate (95% CrI)
Overall	27996	17.44	17.39 (16.80-17.98)
Race and ethnicity			
Hispanic (all races)	8628 (30.8)	18.37	18.70 (17.88-19.51)
Non-Hispanic Black	3125 (11.2)	13.70	13.82 (13.09-14.55)
Non-Hispanic White	13636 (48.7)	17.46	17.46 (16.72-18.21)
Other Non-Hispanic races^b^	2607 (9.3)	20.71	20.51 (19.29-21.74)
Cancer site^c^			
Leukemias	7903 (28.2)	4.96	4.89 (4.67-5.12)
Lymphomas	4002 (14.3)	2.45	2.57 (2.32-2.82)
CNS tumors	4666 (16.7)	2.93	2.76 (2.57-2.95)
Neuroblatomas	1590 (5.7)	1.04	1.00 (0.92-1.08)
Retinoblastomas	504 (1.8)	0.33	0.24 (0.30-0.38)
Renal tumors	1160 (4.1)	0.74	0.66 (0.61-0.71)
Hepatic tumors	532 (1.9)	0.34	0.33 (0.29-0.36)
Bone tumors	1423 (5.1)	0.86	0.92 (0.86-0.98)
Soft tissue sarcomas	1914 (6.8)	1.19	1.20 (1.13-1.27)
Germ cell tumors	1408 (5.0)	0.86	0.85 (0.79-0.90)
Other epithelial neoplasms and melanomas	2695 (9.6)	1.61	1.95 (1.80-2.09)
Other malignant neoplasms	152 (0.5)	0.10	0.09 (0.08-0.11)
Unspecified	47 (0.2)	–	–
Stage at diagnosis^d^			
Early	10111 (36.1)	6.30	6.49 (6.14-6.83)
Late	16989 (60.7)	10.58	10.19 (9.81-10.56)
Other	896 (3.2)	0.56	0.62 (0.35-0.89)

Expected age-adjusted incidence rates for the COVID-19 pandemic (2020-2022) were based on Bayesian age-period-cohort models fit to annual cancer case counts for 2005-2019. Expected rates reported with 95% credible intervals (CrIs).

a Age-adjusted incidence rate per 100 000 population.

b Other non-Hispanic race includes American Indian/Alaska Native, Asian and Pacific Islander, and unknown races.

c Cancer sites defined according to the *International Classification of Childhood Cancer*, Third Edition: leukemias (site group I); lymphomas (site group II); CNS tumors (site group III); neuroblastomas (site group IV); retinoblastomas (site group V); renal tumors (site group VI); hepatic tumors (site group VII); bone tumors (site group VIII); soft tissue sarcomas (site group IX); germ cell tumors (site group X); other epithelial neoplasms (site group XI); other malignant neoplasms (site group XII).

d Stage at diagnosis defined according to the Combined Summary Stage (2004+) variable, where early stage includes localized stage only, late stage includes regional and distant stages, and other includes unknown stage, stage not available, or blank.

Abbreviation: CNS = central nervous system.

The age-adjusted pediatric cancer incidence rate for 2020-2022 was 17.44 per 100 000 population, which was similar to the expected rate of 17.39 (95% CrI = 16.80-17.98) based on pre-pandemic trends. Analyzing annual rates, we found that an initial dip in observed incidence in 2020 (17.23 observed vs 17.67 expected) was offset by slightly (but not significantly) higher than expected rates in 2021 (17.72 vs 17.39) and 2022 (17.39 vs 17.10). Similar patterns were found when stratifying by race/ethnicity and by specific cancer sites, though overall rates of renal tumors were significantly higher than expected across 2020-2022 (0.74 observed vs 0.66 expected [95% CrI = 0.61-0.71]) and overall rates of other epithelial neoplasms and melanomas were significantly lower (1.61 observed vs 1.95 expected [95% CrI = 1.80-2.09]).

A different pattern emerged when pediatric cancer incidence rates were analyzed by stage at diagnosis. For cancers diagnosed at an early stage, the observed annual incidence rate in 2020 was significantly lower than the expected rate (Figure 1). Observed early-stage incidence rates in 2021 and 2022 were also below (though not significantly different from) corresponding expected rate point estimates. In contrast, observed late-stage rates exceeded (but remained statistically similar to) the expected rate point estimates annually. When aggregating results across the COVID-19 pandemic (2020-2022), the observed early-stage incidence rate of 6.30 per 100 000 people occurred within the expected rate range (6.49; 95% CrI = 6.14-6.83), while the observed late-stage pediatric cancer incidence rate of 10.58 was significantly higher than the expected rate of 10.19 (95% CrI = 9.81-10.56). In a sensitivity analysis excluding leukemias (for which >99% of cases are defined as late stage), similar stage-specific results occurred, with the observed rate of late-stage diagnoses during 2020-2022 significantly exceeding the expected rate (5.63 observed vs 5.36 expected [95% CrI = 5.13-5.59]) (Figure S4).

**Figure 1.**
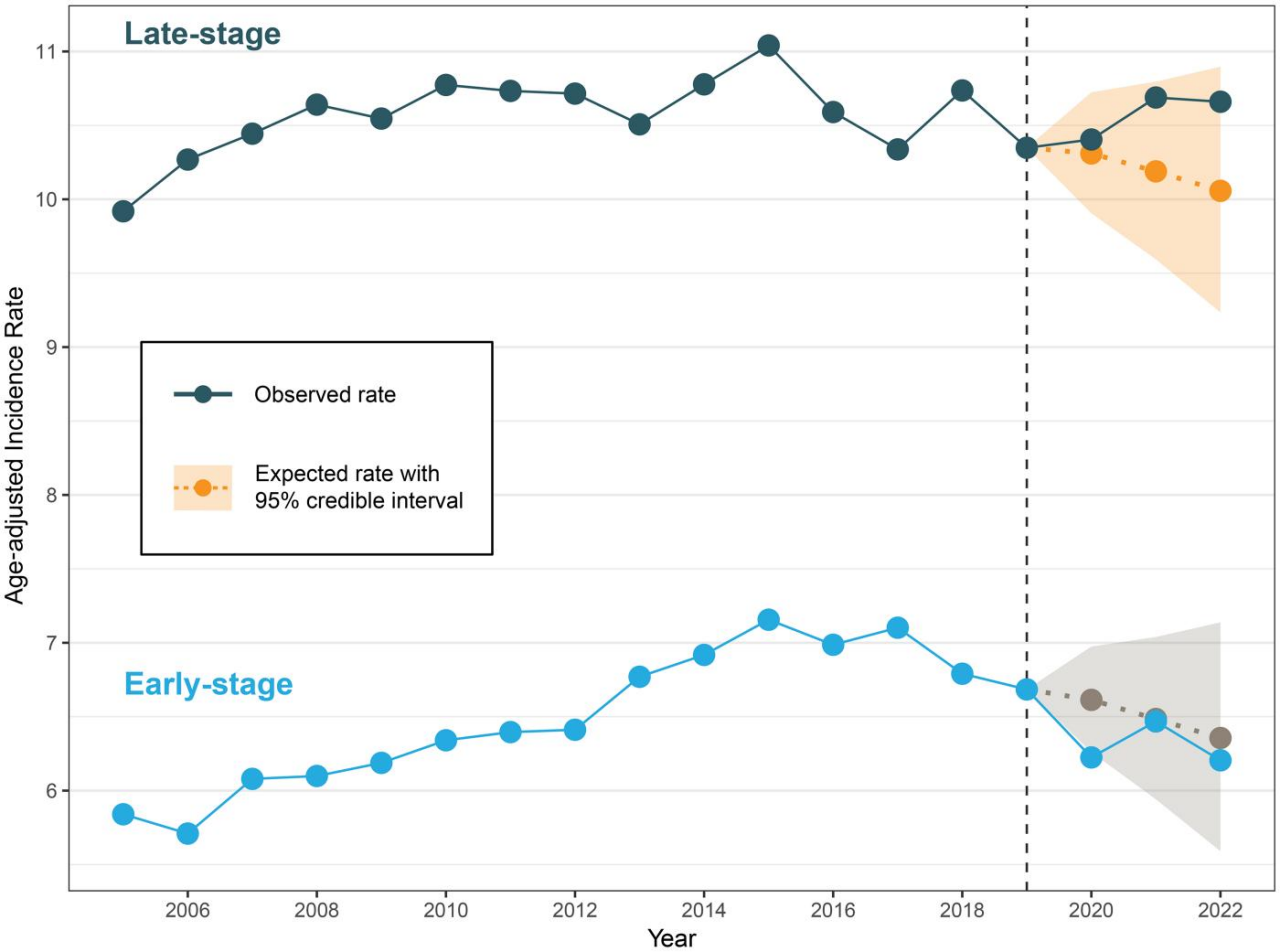
Trends in pediatric cancer incidence rates by stage at diagnosis, 2005-2022. Observed incidence rates for early-stage and late-stage diagnoses displayed for 2005-2022 with expected incidence rates for 2020-2022 as projected by a Bayesian age-period-cohort model.^7^ Stage at diagnosis was defined according to the Combined Summary Stage (2004+) variable, where early stage includes localized stage only and late stage includes regional and distant stages. Expected incidence rates shown with 95% credible intervals.

Site- and stage-specific rates were significantly lower than expected in 2020-2022 for early-stage diagnoses of bone tumors (0.33 observed vs 0.41 expected [95% CrI = 0.36-0.45]) and soft tissue sarcomas (0.55 vs 0.63 [95% CrI = 0.58-0.67]) and late-stage diagnoses of other epithelial neoplasms and melanomas (0.64 vs 0.80 [95% CrI = 0.72-0.88]). Rates were significantly higher than expected for early-stage diagnoses of CNS tumors (2.34 observed vs 2.18 expected [95% CrI = 2.03-2.33]); late-stage diagnoses of germ cell tumors (0.32 vs 0.28 [95% CrI = 0.25-0.31]), soft tissue sarcomas (0.57 vs 0.50 [95% CrI = 0.46-0.54]), and neuroblastomas (0.70 vs 0.63 [95% CrI = 0.58-0.69]); and both early- and late-stage diagnoses of renal tumors (early-stage, 0.30 vs 0.26 [95% CrI = 0.23-0.29]; late-stage, 0.43 vs 0.37 [95% CrI = 0.33-0.41]) (Table S1).

Despite overall pediatric cancer incidence rates aligning with pre-pandemic expectations, we found an increase in, and potential shift toward, later stage at diagnosis during the COVID-19 pandemic. While the absolute difference between observed and expected rates was modest, this corresponded to approximately 625 additional late-stage pediatric cancer diagnoses during 2020-2022 in the NCCR-covered population. Given the association between later stage at diagnosis and more intensive therapy and worsened outcomes, even relatively small population-level increases in later-stage disease may have important clinical implications. Moreover, the appearance of a progressive widening of the gap between annual observed and expected late-stage incidence rate point estimates suggests a concerning trend to monitor coming out of the pandemic period. Potential contributors include reduced in-person primary care visits, fewer school and extracurricular health touchpoints due to closures, and confusion caused by symptom overlap between cancer and COVID-19 infection.^8-13^ Targeted efforts to restore timely evaluation of persistent symptoms in children and to reinforce primary care follow-up are called for to reestablish pre-pandemic patterns of detection. Maintaining access to pediatric diagnostic services should be prioritized during future health-system disruptions.^14^

Study limitations include the lack of full population coverage in our data, assumptions of the predictive modeling approach, the ecological design, and small numbers that precluded more granular analyses for some population subgroups.^7^ The potential exists for underreporting of cases in recent years, which could lead to underestimation of observed incidence rates. Because underreporting would bias incidence rates downward, the observation of rates meeting or exceeding expected values, particularly for late-stage disease, may be conservative. However, uncertainty regarding whether any potential underreporting differed by stage at diagnosis may affect interpretation of an observed shift toward later-stage diagnoses. The appearance of a widening gap between annual observed and expected late-stage incidence rate point estimates is based on only 3 years of data; further analysis is called for as data become available to determine whether this pattern persists over time. Coding changes in the ICCC-3 may have impacted classification of cases over time, though most changes took place in site subgroups or stages more granular than used in this study. Nevertheless, our use of high-quality, population-based cancer data covering approximately 73% of the US population aged 0-39 years and a well-attested modeling approach provides new information about pediatric cancer diagnoses during the COVID-19 pandemic.

## Supplementary Material

pkag067_Supplementary_Data

## Data Availability

Data is available through a restricted use agreement with the National Cancer Institute.

## References

[1] Heffernan ME , Macy ML. Trends in mental and physical health among youths. JAMA Pediatr. 2025;179:683-685. 10.1001/jamapediatrics.2025.0556 40257756 PMC12013346

[2] Ford ND , Vahratian A, Pratt CQ, Yousaf AR, Gregory CO, Saydah S. Long COVID prevalence and associated activity limitation in US children. JAMA Pediatr. 2025;179:471-473. 10.1001/jamapediatrics.2024.6206 39899316 PMC11791766

[3] Semprini J , Mobley EM. Understanding the 2020 pediatric cancer deficit: insights from the National Childhood Cancer Registry. Pediatr Blood Cancer. 2024;71:e31345. 39367593 10.1002/pbc.31345 PMC12712806

[4] Siegel DA , Kava CM, Spector LG, et al Pediatric and young adult cancer incidence in the United States during the COVID‐19 pandemic. Pediatr Blood Cancer. 2025;72:e31976. 40836499 10.1002/pbc.31976 PMC12416144

[5] Steliarova‐Foucher E , Stiller C, Lacour B, Kaatsch P. International classification of childhood cancer, third edition. Cancer. 2005;103:1457-1467. 10.1002/cncr.20910 15712273

[6] Riebler A , Held L. Projecting the future burden of cancer: Bayesian age–period–cohort analysis with integrated nested Laplace approximations. Biometrical J. 2017;59:531-549. 10.1002/bimj.201500263 28139001

[7] Burus T , Kim U, Rose J, Koroukian SM, Lang Kuhs KA. A cross-sectional assessment of US cancer diagnoses during the COVID-19 pandemic. Cancer Epidemiol. 2025;99:102944. 10.1016/j.canep.2025.102944 41108855

[8] Moreira DC , Millen GC, Sands S, Kearns PR, Hawkins DS. The care of children with cancer during the COVID-19 pandemic. Am Soc Clin Oncol Edu Book. 2021;41:e305-e314. 10.1200/EDBK_321497 33989020

[9] Batioja K , Elenwo C, Hartwell M. Disparities in pediatric medical and childcare disruption due to COVID-19. JAMA Pediatr. 2023;177:432-434. 10.1001/jamapediatrics.2022.6130 36806461 PMC9941966

[10] Offenbacher R , Knoll MA, Loeb DM. Delayed presentations of pediatric solid tumors at a tertiary care hospital in the Bronx due to COVID‐19. Pediatr Blood Cancer. 2021;68:e28615. 10.1002/pbc.28615 32725878

[11] Teasdale CA , Borrell LN, Shen Y, et al Missed routine pediatric care and vaccinations in US children during the first year of the COVID-19 pandemic. Prev Med. 2022;158:107025. 10.1016/j.ypmed.2022.107025 35318030 PMC8933962

[12] Ding Y , Ramakrishna S, Long AH, et al Delayed cancer diagnoses and high mortality in children during the COVID‐19 pandemic. Pediatr Blood Cancer. 2020;67:e28427. 10.1002/pbc.28427 32588960 PMC7361231

[13] Ozluk P , Romine J, Sylwestrzak G, Hamad R. Effect of school reopenings on children’s mental health during COVID-19: quasi-experimental evidence from California. Epidemiology (Fairfax). 2026;37:257-267. 10.1097/EDE.0000000000001930 PMC12824883 41364631

[14] Graetz D , Agulnik A, Ranadive R, et al Global effect of the COVID-19 pandemic on paediatric cancer care: a cross-sectional study. Lancet Child Adolesc Health. 2021;5:332-340. 10.1016/S2352-4642(21)00031-6 33675698 PMC7929816

