# Supplementary material for: Pandemic-era increases in late-stage pediatric cancer diagnoses, 2020-2022
Source: JNCI Cancer Spectr. 2026 Jun 22;10(4):pkag067. doi: 10.1093/jncics/pkag067 (PMC13355589; doi:10.1093/jncics/pkag067)
Supplement: pkag067_Supplementary_Data [file pkag067_supplementary_data.docx]

**Supplementary Online Content**

**Figure S1.** Trends in pediatric cancer incidence rates by stage at diagnosis (excluding leukemias), 2005-2022

**Figure S2.** Trends in pediatric cancer incidence rates by stage at diagnosis (excluding leukemias), 2005-2022

**Figure S3.** Trends in pediatric cancer incidence rates by stage at diagnosis (excluding leukemias), 2005-2022

**Figure S4.** Trends in pediatric cancer incidence rates by stage at diagnosis (excluding leukemias), 2005-2022

**Table S1.** Observed versus expected pediatric cancer incidence rates for the COVID-19 pandemic by site and stage at diagnosis

This supplementary material has been provided by the authors to give readers additional information about their work.

**Figure S1. Diagnostic plots for overall pediatric cancer incidence model, 2005-2022 (1).** (1) Posterior predictive fit plot, and (2) residual plot for Bayesian Age-Period-Cohort model fit to all pediatric cancer cases diagnosed between 2005 and 2019.


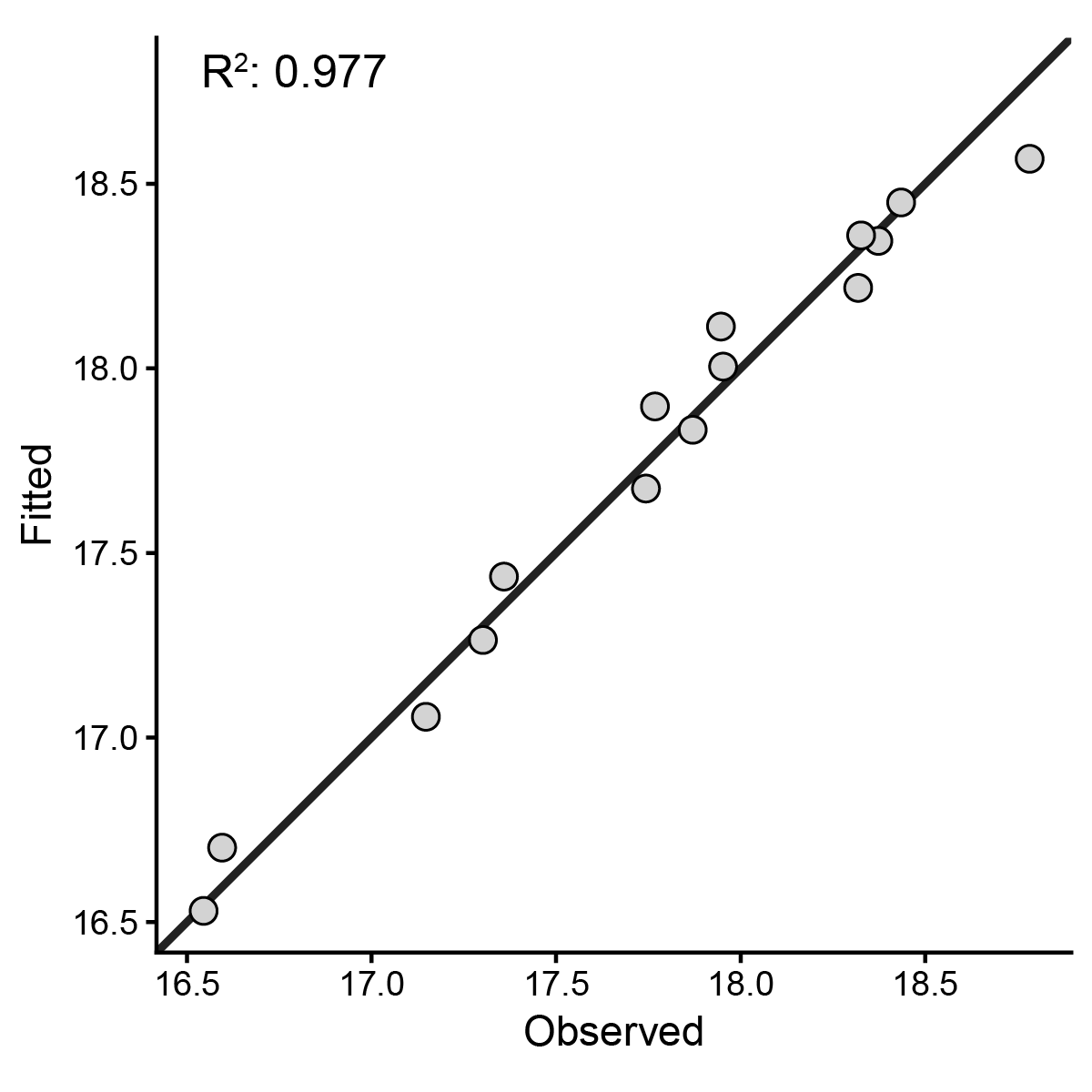
(1)


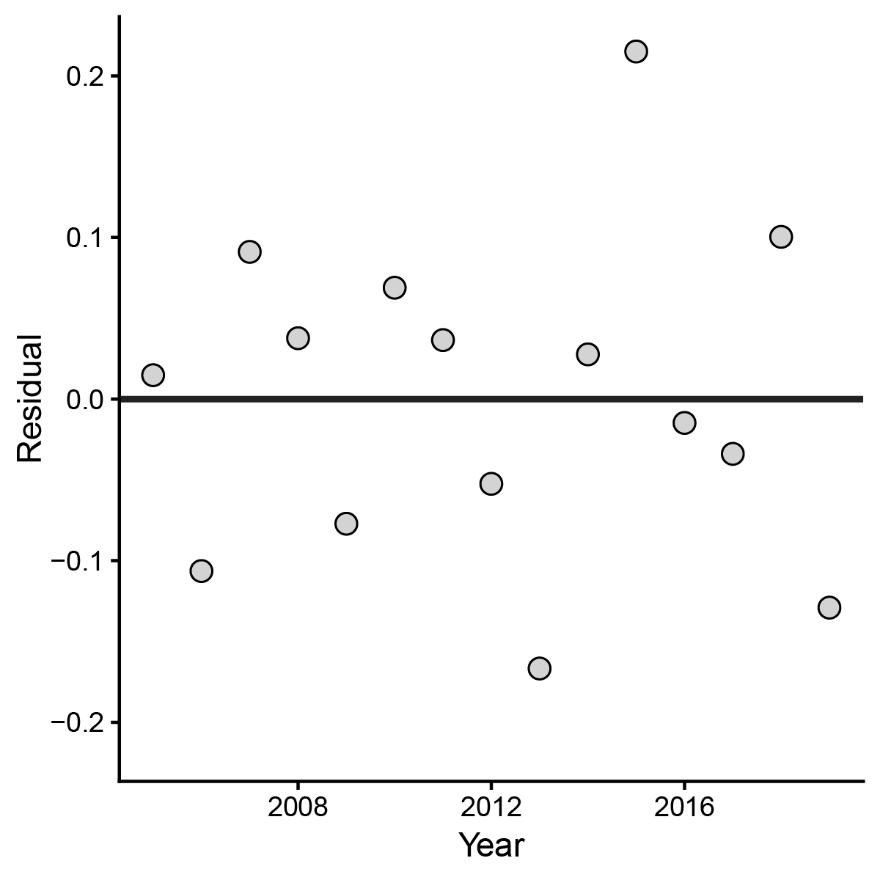
(2)

**Figure S2. Diagnostic plots for early-stage pediatric cancer incidence model, 2005-2022 (1).** (1) Posterior predictive fit plot, and (2) residual plot for Bayesian Age-Period-Cohort model fit to early-stage pediatric cancer cases diagnosed between 2005 and 2019.


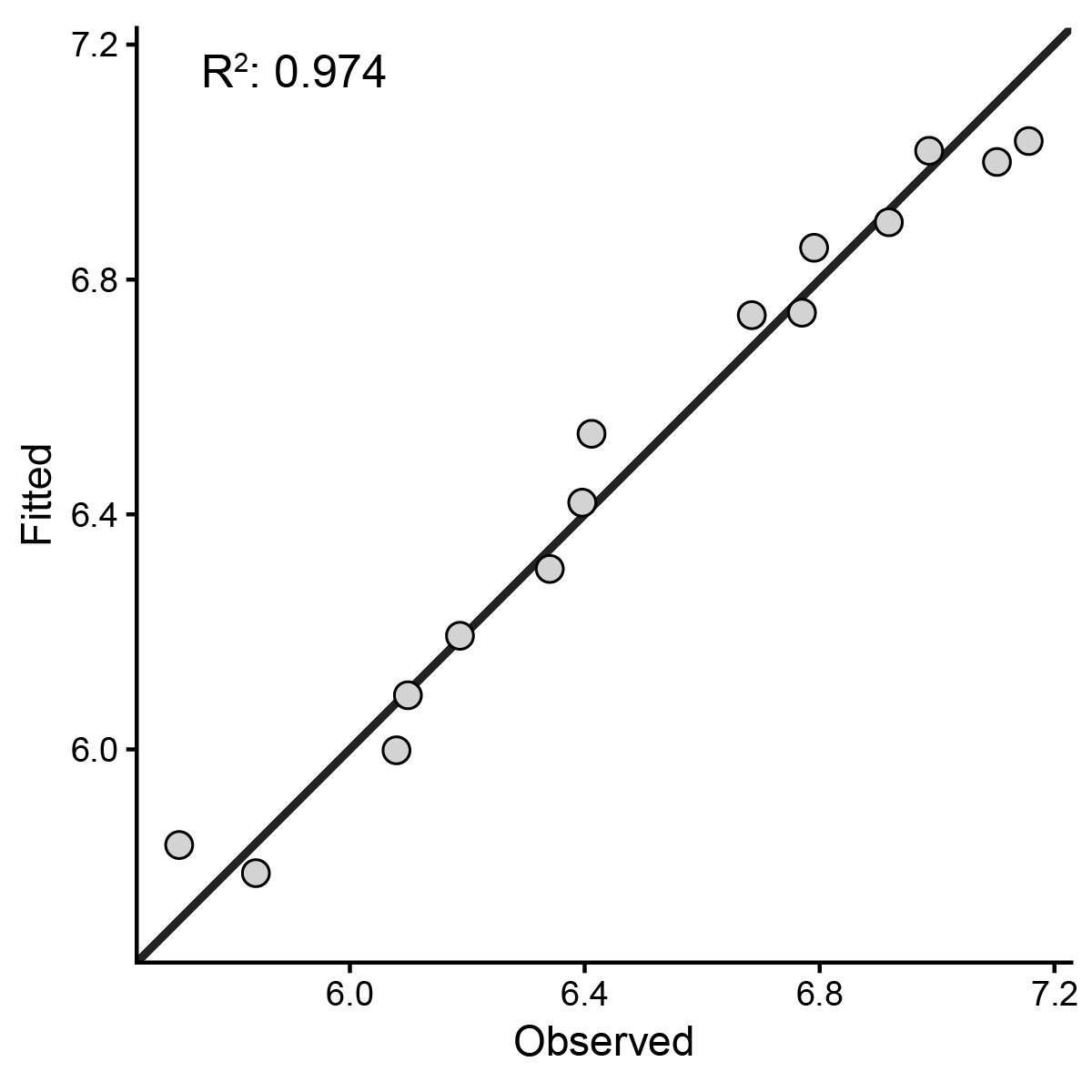
(1)


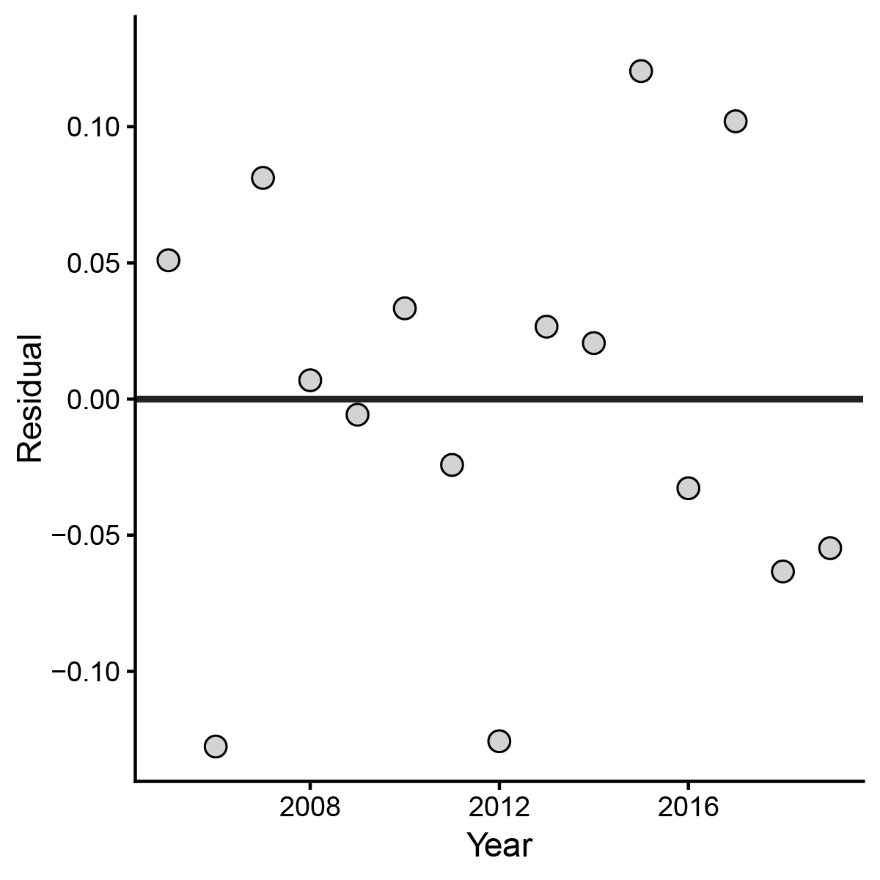
(2)

**Figure S3. Diagnostic plots for late-stage pediatric cancer incidence model, 2005-2022 (1).** (1) Posterior predictive fit plot, and (2) residual plot for Bayesian Age-Period-Cohort model fit to late-stage pediatric cancer cases diagnosed between 2005 and 2019.


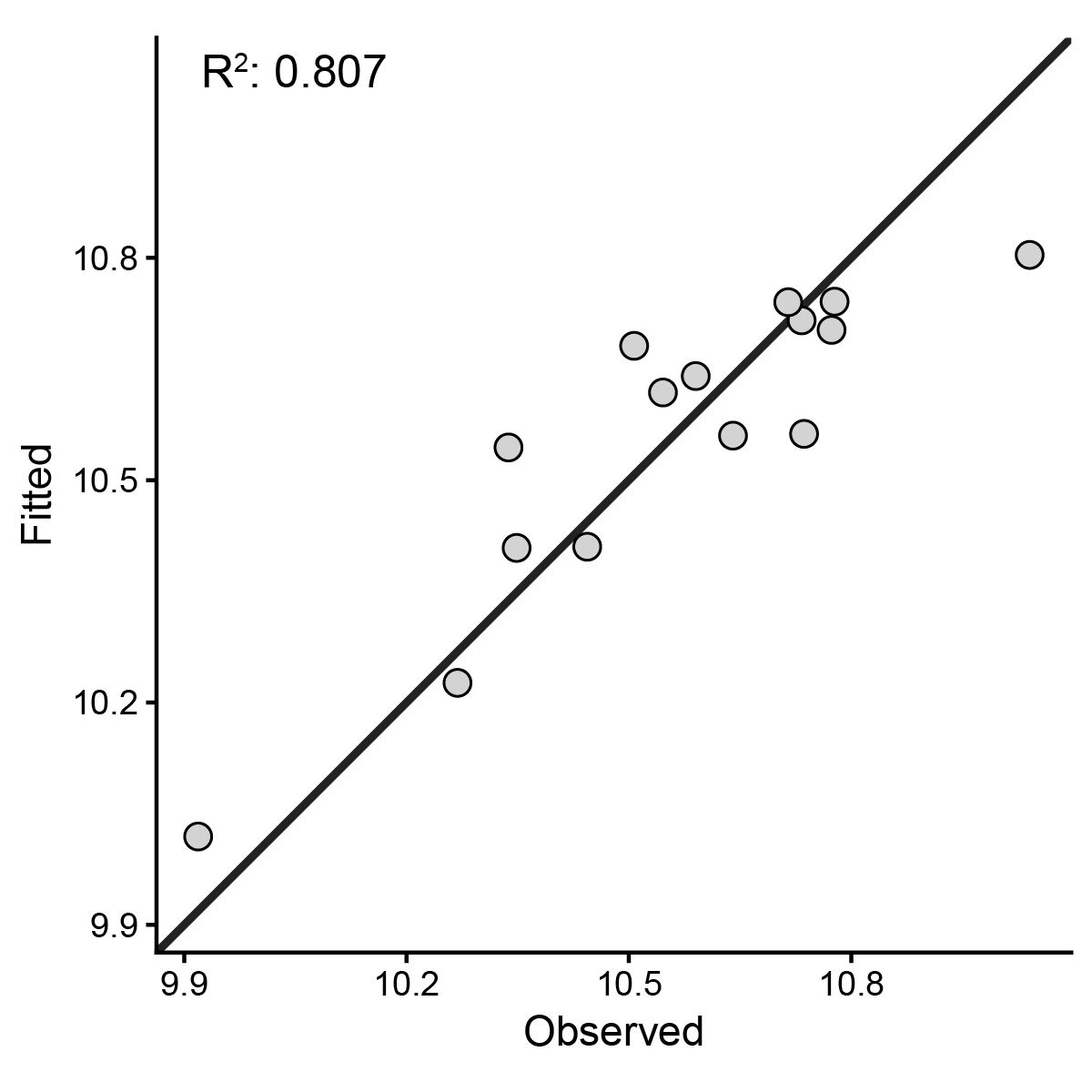
(1)


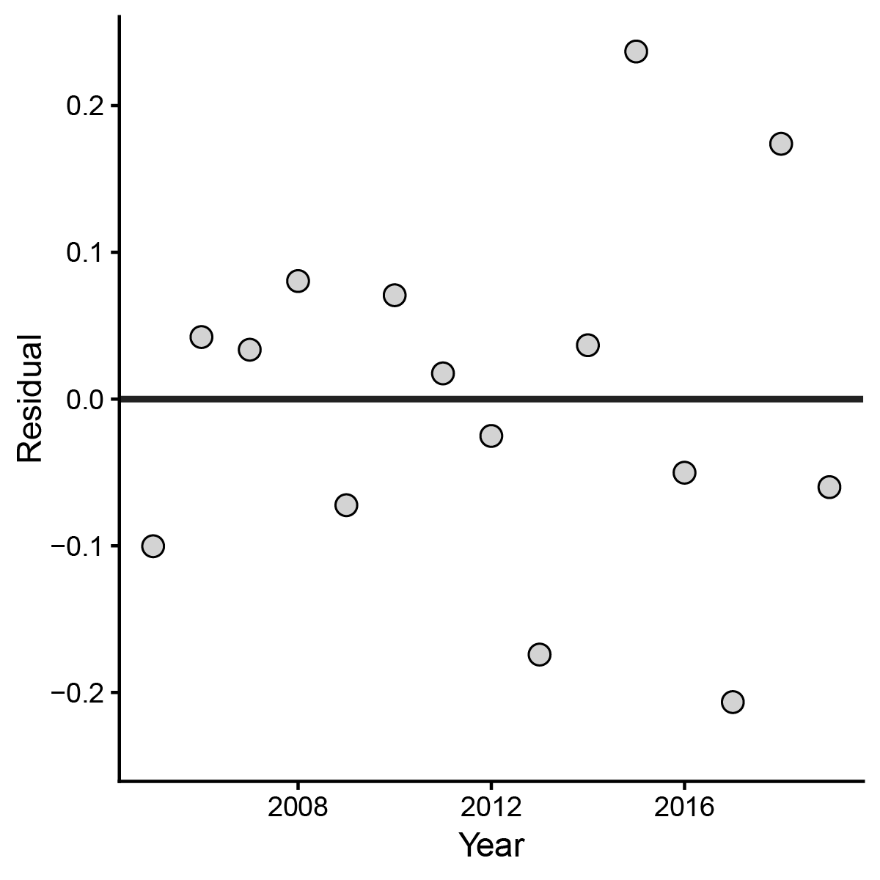
(2)

**Figure S4. Trends in pediatric cancer incidence rates by stage at diagnosis (excluding leukemias), 2005-2022 (1).** Observed incidence rates for early- and late-stage diagnoses displayed for 2005-2022 with expected incidence rates for 2020-2022 as projected by Bayesian Age-Period-Cohort model (2). Diagnoses of leukemia were excluded due to >99% of cases being diagnosed at a late stage. Stage at diagnosis was defined according to the Combined Summary Stage (2004+) variable, where early stage includes localized stage only and late stage includes regional and distant stages. Expected incidence rates shown with 95% credible intervals.

**
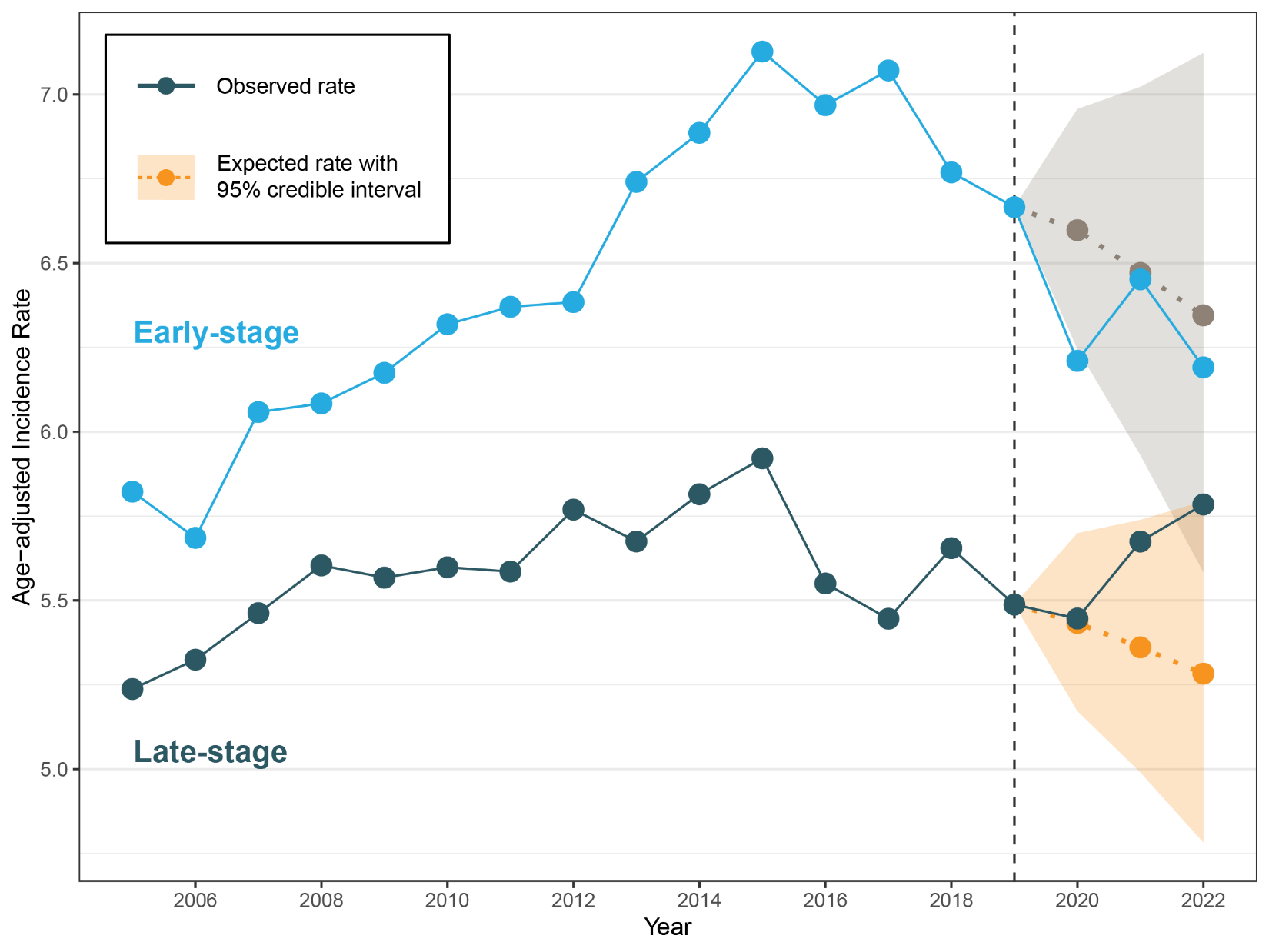
**

**Table S1. Observed versus expected pediatric cancer incidence rates for the COVID-19 pandemic by site and stage at diagnosis (1).** Expected age-adjusted incidence rates for the COVID-19 pandemic (2020-2022) were based on Bayesian Age-Period-Cohort models fit to annual cancer case counts for 2005-2019 (2). Expected rates reported with 95% credible intervals (CrI).

| **Site^a^** | **Stage^b^** | **Count, N** | **Observed Rate^c^** | **Expected Rate (95% CrI)** |
| --- | --- | --- | --- | --- |
| Leukemias | Early | 8 | 0.00 | 0.02 (0.01-0.03) |
|  | Late | 7886 | 4.95 | 4.86 (4.63-5.08) |
|  | Other | 9 | - | - |
| Lymphomas | Early | 866 | 0.53 | 0.74 (0.51-0.96) |
|  | Late | 2962 | 1.81 | 1.71 (1.59-1.83) |
|  | Other | 174 | - | - |
| CNS Tumors | Early | 3727 | 2.34 | 2.18 (2.03-2.33) |
|  | Late | 748 | 0.47 | 0.47 (0.43-0.52) |
|  | Other | 191 | - | - |
| Neuroblastomas | Early | 447 | 0.29 | 0.3 (0.26-0.33) |
|  | Late | 1068 | 0.70 | 0.63 (0.58-0.69) |
|  | Other | 75 | - | - |
| Retinoblastomas | Early | 413 | 0.27 | 0.28 (0.25-0.32) |
|  | Late | 59 | 0.04 | 0.04 (0.03-0.05) |
|  | Other | 32 | - | - |
| Renal Tumors | Early | 466 | 0.30 | 0.26 (0.23-0.29) |
|  | Late | 666 | 0.43 | 0.37 (0.33-0.41) |
|  | Other | 28 | - | - |
| Hepatic Tumors | Early | 285 | 0.19 | 0.18 (0.16-0.21) |
|  | Late | 223 | 0.14 | 0.13 (0.11-0.15) |
|  | Other | 24 | - | - |
| Bone Tumors | Early | 547 | 0.33 | 0.41 (0.36-0.45) |
|  | Late | 821 | 0.50 | 0.47 (0.43-0.51) |
|  | Other | 55 | - | - |
| Soft Tissue Sarcomas | Early | 888 | 0.55 | 0.63 (0.58-0.67) |
|  | Late | 920 | 0.57 | 0.5 (0.46-0.54) |
|  | Other | 106 | - | - |
| Germ Cell Tumors | Early | 819 | 0.50 | 0.52 (0.48-0.57) |
|  | Late | 526 | 0.32 | 0.28 (0.25-0.31) |
|  | Other | 63 | - | - |
| Other Epithelial Neoplasms and Melanomas | Early | 1515 | 0.91 | 0.99 (0.77-1.21) |
|  | Late | 1072 | 0.64 | 0.8 (0.72-0.88) |
|  | Other | 91 | - | - |
| Other Malignant Neoplasms | Early | 75 | 0.05 | 0.05 (0.04-0.06) |
|  | Late | 29 | 0.02 | 0.02 (0.02-0.03) |
|  | Other | 48 | - | - |

Abbreviations: CNS = central nervous system

^a^ Cancer sites defined according to the International Classification of Childhood Cancer, Third Edition: leukemias (site group I); lymphomas (site group II); CNS tumors (site group III); neuroblastomas (site group IV); retinoblastomas (site group V); renal tumors (site group VI); hepatic tumors (site group VII); bone tumors (site group VIII); soft tissue sarcomas (site group IX); germ cell tumors (site group X); other epithelial neoplasms (site group XI); other malignant neoplasms (site group XII).
^b^ Stage at diagnosis defined according to the Combined Summary Stage (2004+) variable, where early stage includes localized stage only, late stage includes regional and distant stages, and other includes unknown stage, stage not available, or blank.
^c^ Age-adjusted incidence rate per 100,000 population.

**References**

1. SEER*Stat Database. NCCR Incidence Data (ages 0-39), 25 States and Seattle, Dec 2024 NAACCR Sub (1997-2022) (which includes data from CDC’s National Program of Cancer Registries (NPCR), CCR’s Provincial and Territorial Registries, and the NCI’s Surveillance, Epidemiology and End Results (SEER) Registries). Registries included: AR, CO, CA, CT, FL, GA, HI, ID, IL, IA, KY, LA, MA, MI, MN, MO, NJ, NY, NM, NY, OR, PA, TN, TX, WI, UT, and Seattle.

2. Burus T, Kim U, Rose J, Koroukian SM, Lang Kuhs KA. A cross-sectional assessment of US cancer diagnoses during the COVID-19 pandemic. *Cancer Epidemiology*. 2025;99:102944. doi:10.1016/j.canep.2025.102944
